# Case Report: First case of low-grade myofibroblastic sarcoma of the vulva during pregnancy

**DOI:** 10.3389/fonc.2025.1577068

**Published:** 2025-06-10

**Authors:** San Zhu, Yan Luo, Ce Bian, Yaoyao Zhang, Lingyun Yang

**Affiliations:** ^1^ Department of Gynecology and Obstetrics, West China Second University Hospital, Sichuan University, Chengdu, China; ^2^ West China School of Medicine, Sichuan University, Chengdu, China; ^3^ Key Laboratory of Birth Defects and Related Diseases of Women and Children, Ministry of Education, Sichuan University, Chengdu, China; ^4^ Department of Gynecology, Hospital of Chengdu University of Traditional Chinese Medicine, College of Clinical Medicine, Chengdu University of Traditional Chinese Medicine, Chengdu, China

**Keywords:** low-grade myofibroblastic sarcoma, rare tumor, vulvar, pregnancy, case report

## Abstract

Low-grade myofibroblastic sarcoma (LGMFS) of the vulva is exceptionally rare, with only two prior cases reported. We present the third documented case globally and the first occurring during pregnancy, highlighting diagnostic and therapeutic challenges in this unique clinical scenario. A 34-year-old woman presented with a recurrent vulvar mass initially misdiagnosed as angiomyofibroblastoma. The lesion reappeared asymptomatically during pregnancy and was conservatively managed with ultrasound surveillance, followed by term cesarean delivery to mitigate perineal trauma risks. Postpartum evaluation revealed a 3.7 cm T2-hyperintense nodule on MRI. Although intraoperative frozen sections suggested benign margins, definitive histopathology and molecular studies (CD34+/SMA+; FISH-negative for COL1A1::PDGFB fusion and MDM2 amplification) confirmed LGMFS. Radical vulvectomy with 2 cm margins achieved disease-free survival at 17 months without adjuvant therapies. This case underscores that LGMFS may recur silently during pregnancy, necessitating rigorous histopathological re-evaluation of prior benign diagnoses. Multidisciplinary coordination enabled safe deferral of definitive surgery until postpartum without compromising outcomes, while radical excision alone proved curative, reflecting the tumor’s indolent biology. Our findings establish the first pragmatic framework for managing vulvar LGMFS in pregnancy, emphasizing tailored surgical planning over routine adjuvant interventions.

## 1 Introduction

Low-grade myofibroblastic sarcoma (LGMFS) is an exceptionally rare and often misdiagnosed tumor, accounting for approximately 0.6% of all malignant soft tissue tumors (1). The actual incidence of this tumor is likely underestimated due to the inherent challenges in achieving an accurate diagnosis (2). The tumor was first comprehensively characterized by Mentzel et al. in 1998 (3), and subsequently classified as a distinct group of soft tissue and bone tumors by the World Health Organization in 2002 (4). Despite its typically locally aggressive behavior, LGMFS generally has a relatively favorable prognosis, with low rates of metastasis and a tendency for local recurrence (5, 6). A recent population-based study in the United States reported a five-year overall survival rate of 71.6% for LGMFS patients (7). LGMFS most commonly affects adults, with a slight male predominance, though gender distribution is not well-established due to the rarity of the condition and limited sample sizes in studies (2, 8). It is a rare mesenchymal tumor and frequently located within subcutaneous and deep soft tissues (9, 10). While it can develop in almost any region of the body, it predominantly occurs in the head and neck, especially in areas such as the oral cavity and tongue (2, 11, 12). Only two cases involving the vulva have been reported to date (13, 14), and there is no documented case related to pregnant women. Due to the rarity and plasticity of myofibroblasts, the diagnosis of LGMFS can be challenging and is often subject to controversy, with the potential for misdiagnosis as a benign tumor (15). Moreover, the optimal treatment strategy for LGMFS remains undefined, particularly in cases complicated by pregnancy, where specific diagnostic and therapeutic guidelines are lacking. To raise awareness of this tumor in the lower female genital tract and to emphasize its clinical presentation, differential diagnoses, natural history, and long-term prognosis, we present the third reported case of vulvar LGMFS and, for the first time, provide insights into its manifestation during pregnancy.

## 2 Case report

**Figure 1:**
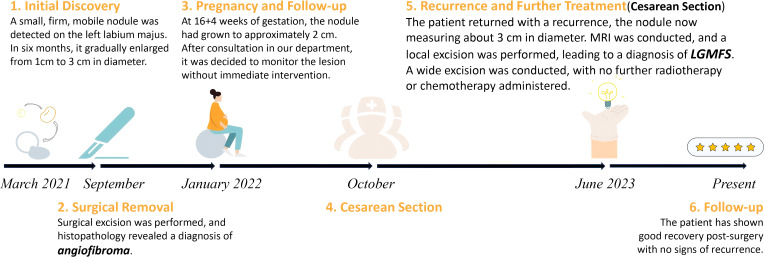
Timeline of the diagnosis and management of the case, illustrating initial discovery, surgical excision, monitoring, recurrence, and follow-up. LGMFS, low-grade myofibroblastic sarcoma; MRI, magnetic resonance imaging.

A 34-year-old Chinese woman was admitted to our hospital due to a palpable and non-tender vulvar mass (**Figure 1**). She reported no abdominal pain, abnormal vaginal bleeding, or discharge. Her family medical history was unremarkable. More than two years prior, she had experienced a painless vulvar mass in the same location. At that time, the local hospital diagnosed it as a Bartholin gland cyst and performed a “cyst excision”. Postoperative histopathological examination suggested angiomyofibroblastoma, and the patient was advised to have regular follow-ups without further treatment. Approximately six months later, she became pregnant and, at around four months of gestation, noticed the recurrence of the vulvar mass at the same location, which was about 2 cm in diameter. At this point(at about 4 months of gestation), she sought consultation at our hospital. A multidisciplinary consultation was conducted, involving gynecology, obstetrics, pathology, and imaging specialists. The newly developed vulvar mass was located at the same site as the previous lesion, strongly suggesting a relapse of the original condition. Furthermore, our pathology department reviewed the initial excision specimen from the local hospital. Despite the limited and fragmented tissue samples, the diagnosis of angiomyofibroblastoma was confirmed. Imaging studies also showed no signs of aggressive growth or metastasis, reinforcing our suspicion of a recurrence of angiomyofibroblastoma. Given the uncertain nature of the perineal tumor, a repeated biopsy during pregnancy was not prioritized due to concerns about potential tumor dissemination, metastasis, or uncontrollable bleeding. The obstetrics team also determined that there was no immediate indication for pregnancy termination based on the current diagnosis. After the comprehensive discussion, we recommended regular monitoring, with plans to address the vulvar mass after the delivery, provided no progression occurred. Fortunately, the patient was able to carry the pregnancy to term without significant enlargement of the tumor or any other abnormal symptoms. When labor approached, a cesarean section was performed to avoid potential complications during vaginal delivery. Although the perineal tumor measured only about 2 cm and was most likely a recurrence of angiomyofibroblastoma, malignancy could not be completely ruled out. Concerns about perineal congestion, edema, tearing, and the potential need for episiotomy during labor-any of which could have led to rapid tumor progression, dissemination, or metastasis-justified the decision for cesarean delivery. The patient successfully underwent a cesarean section and delivered a healthy baby. Six months postpartum, after completing breastfeeding, the patient returned to our hospital(this admission), reporting no significant increase in the size of the mass during pregnancy, and no pain, abnormal vaginal bleeding, or discharge.

**Figure 2:**
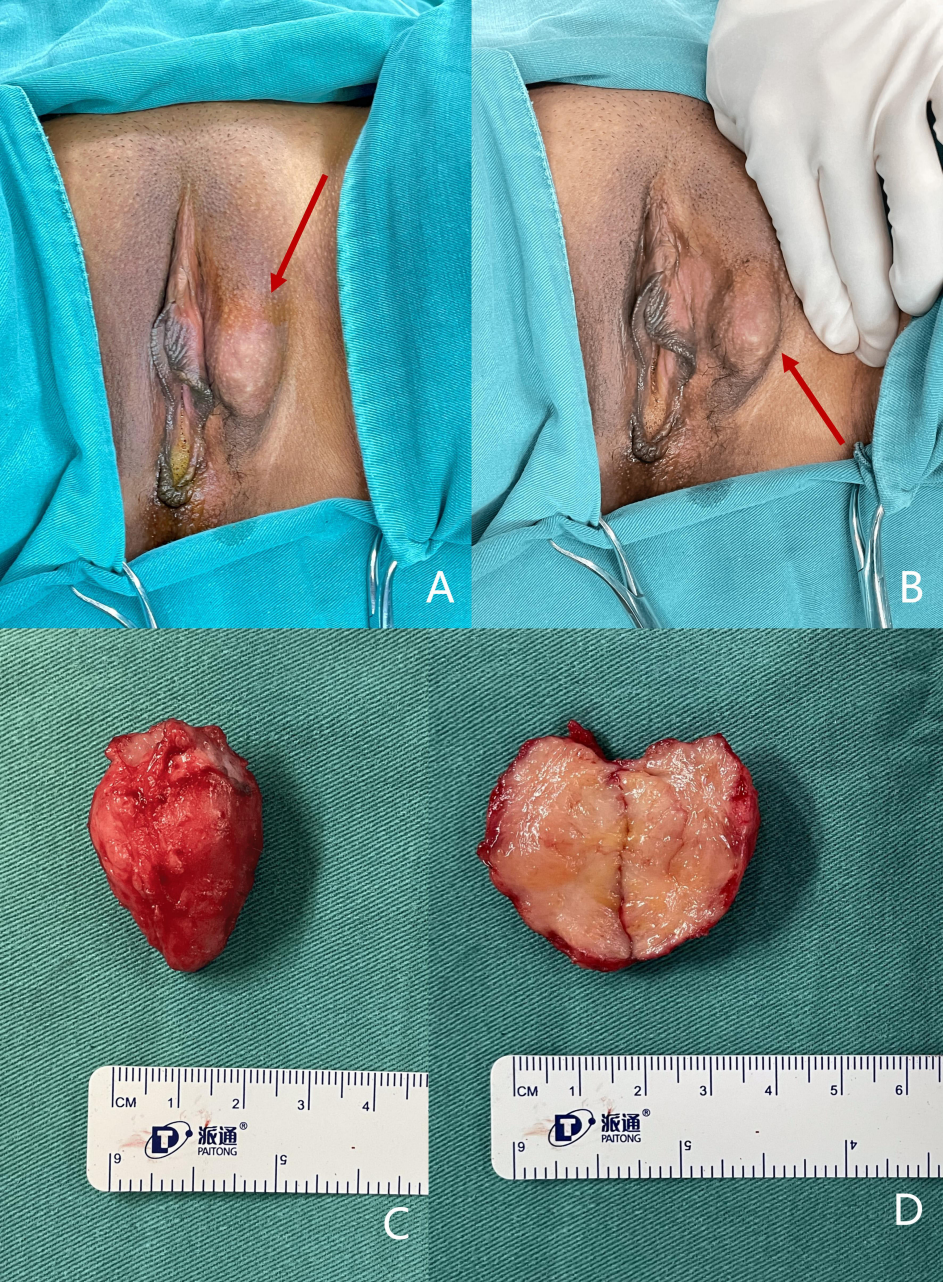
**(A, B)** Gross appearance of the vulvar mass (indicated by the red arrow) located on the lower middle portion of the left labium majus, measuring approximately 2 cm in its longest dimension. **(C, D)** Gross appearance of the excised LGMFS **(C)** and its cross-section **(D)**. **(C)** The tumor is firm with an irregular surface and well-defined borders. **(D)** The cross-section reveals a white, fibrous texture without evidence of hemorrhage or necrosis.

**Figure 3:**
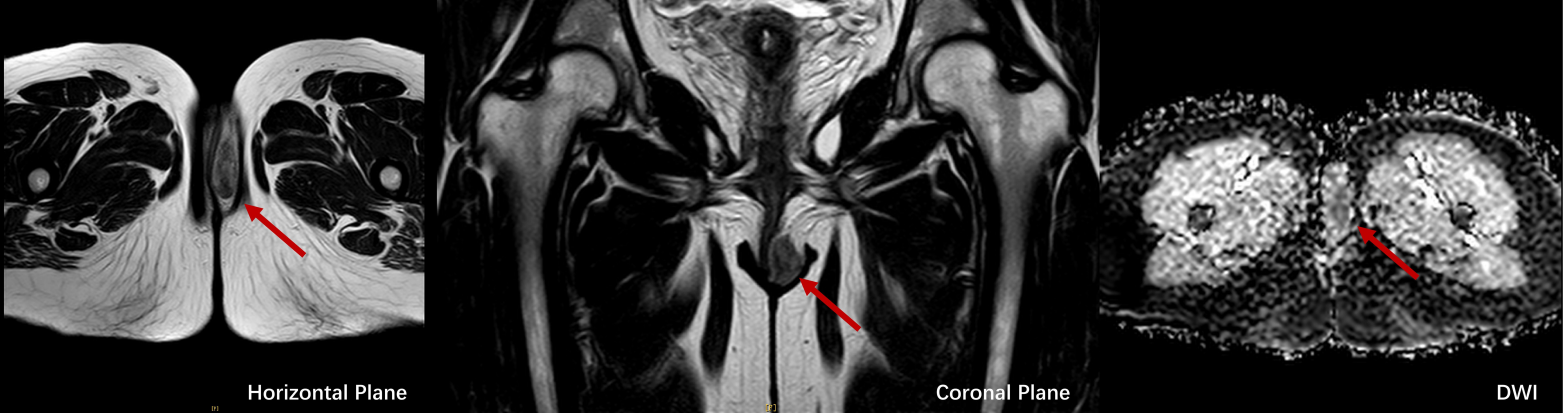
MRI images of LGMFS, indicated by red arrows, in horizontal (left), coronal (middle), and diffusion-weighted imaging (DWI, right) planes, showcasing restricted diffusion and well-defined tumor margins.

Upon this admission, physical examination revealed a spindle-shaped, solid mass approximately 3 × 2 × 2 cm in size, located subcutaneously at the lower end of the left labia majora (**Figures 2A, B**). The mass was hard, with an irregular surface and relatively well-defined borders. MRI showed an abnormal signal nodule in the left labia majora, measuring about 3.7 × 1.5 × 2.2 cm (anteroposterior × transverse × craniocaudal diameters). The mass exhibited a slightly high signal on T2-weighted imaging (T2WI), an isointense signal on T1-weighted imaging (T1WI), restricted diffusion, clear borders, and significant enhancement on contrast scans (**Figure 3**). Subsequently, the patient underwent a simple vulvar mass excision for a diagnosis of angiofibroblastoma. The excised mass appeared white and fibrous on the cut surface, with no hemorrhage or necrosis observed (**Figures 2C, D**). The intraoperative frozen section confirmed that the surgical margins were free of disease involvement.

**Figure 4:**
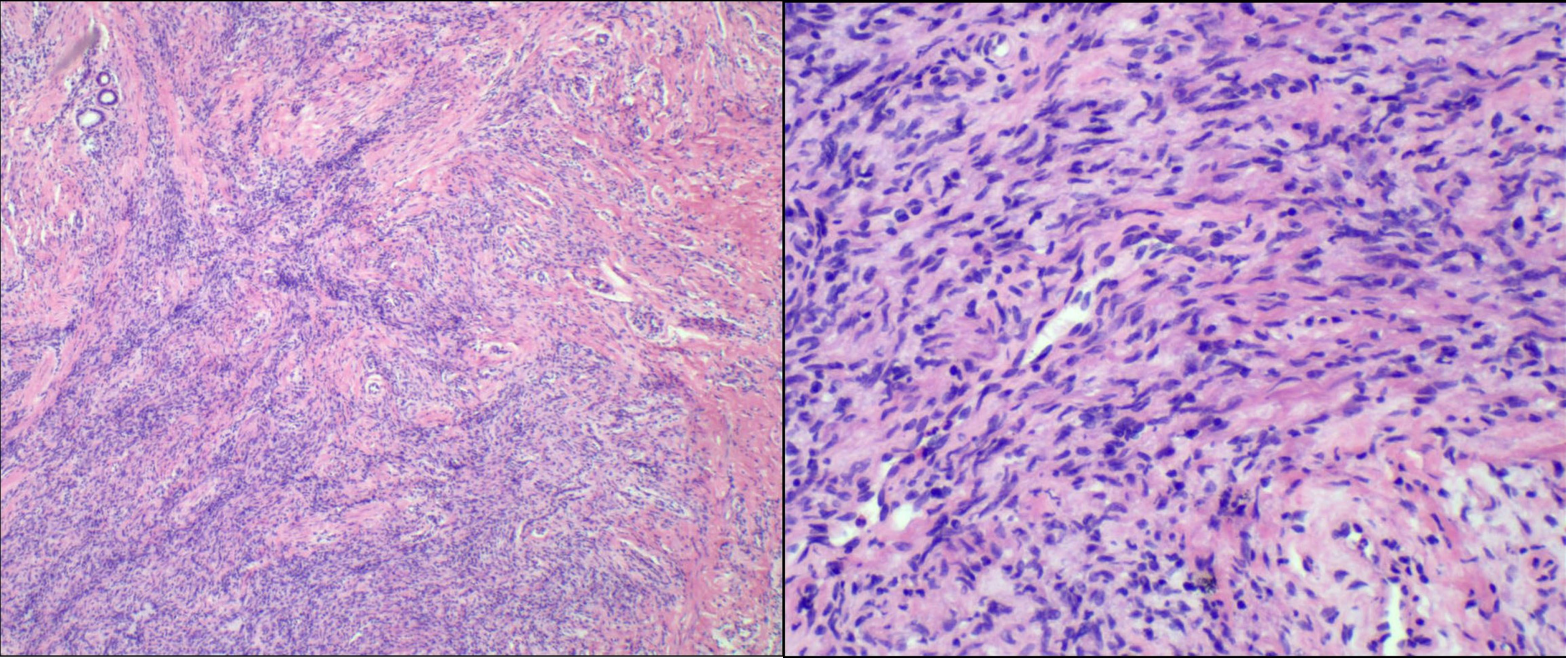
Microscopic view of the tumor showing an absence of a capsule and an indistinct boundary with surrounding tissues. Cellular proliferation with prominent mitotic activity is easily identifiable in certain regions.

However, final paraffin-embedded histopathological examination revealed a spindle cell tumor exhibiting invasive growth, infiltrating the surrounding adipose tissue. The cells showed atypia and mitotic figures were present (**Figure 4**). Immunohistochemical staining results were as follows: CD34 (partial +), CD10 (-), SMA (focal +), TRK (Pan) (-), S100 (-), H3 K27Me3 (no loss), Desmin (-), CDK4 (-), p16 (partial -), STAT6 (-), p53 (partial +), EMA (-), TLE1 (-), and Ki-67 (MIB-1) showing 5% positivity. Fluorescence *in situ* hybridization (FISH) was performed to refine the diagnosis, which showed no evidence of COL1A1::PDGFB gene fusion, PDGFB gene (22q13) translocation, or MDM2 gene (12q15) amplification. Based on the comprehensive evaluation, the diagnosis was determined to be LGMFS.

Following the new pathological diagnosis, the patient underwent a second surgery for radical local excision (RLE). This involved a radical left vulvectomy with a 2 cm margin around the tumor and a 2 cm depth of tissue at the base. The excision extended to the inferior layer of the urogenital diaphragm, in accordance with recommendations for vulvar tumors, despite no evidence of recurrence. Inguinal lymph node dissection and adjuvant therapy were not performed, given the low-grade nature of the tumor and negative surgical margins. Seventeen months postoperatively, the patient remains free of local or metastatic recurrence, as confirmed by physical examination and computed tomography (CT) scans of the chest, abdomen, and pelvis.

## 3 Discussion

### 3.1 Current landscape of vulvar LGMFS: a rare entity with unmet needs

Low-grade myofibroblastic sarcoma (LGMFS) is a rare soft tissue sarcoma that most commonly occurs in the head and neck region. To date, only two cases of vulvar LGMFS have been reported. In the first case, a 4 × 3 cm vulvar mass was treated with wide local excision, and no recurrence was observed during a 14-month follow-up. Notably, this patient did not receive adjuvant therapy, emphasizing the importance of achieving complete surgical excision to prevent recurrence, given the tumor’s locally invasive yet low-grade nature. Similarly, the second case involved a more extensive radical local excision to ensure clear surgical margins, and the patient remained disease-free for 72 months post-surgery, without any adjuvant treatment. Both cases highlight the significance of obtaining clear surgical margins to prevent local recurrence, and the decision to forgo adjuvant therapy in favor of close postoperative surveillance. However, there is no available literature addressing the management of vulvar LGMFS during pregnancy and this is the first. The unique clinical presentation and management strategy employed in this case highlight the need for a more standardized approach to such rare conditions in pregnant patients. We conducted a narrative review of the existing case reports of LGMFS (published literature on vulvar neoplasms was identified in MEDLINE, Cochrane Library, Web of Science, and EMBASE from inception to 2024. MeSH key words used included ‘‘leiomyosarcoma’’, ‘‘myofibroblast’’, ‘‘sarcoma’’, and ‘‘vulvar neoplasms’’), focusing on clinical presentation, diagnosis, treatment, and prognosis, aiming to enhance the clinical management of this rare tumor and provide clinicians with a more informed basis for making treatment decisions, especially in complex cases like those occurring during pregnancy.

### 3.2 Management challenges in vulvar LGMFS: a multidisciplinary approach

The diagnosis and management of LGMFS itself are highly challenging, particularly when it arises in the vulva, a rare site for this tumor. The diagnosis of LGMFS is challenging due to its lack of specific clinical manifestations. As seen in our case, the patient only exhibited a painless vulvar mass, significantly increasing the difficulty of diagnosis. The differential diagnosis of vulvar masses is challenging due to the wide variety of potential conditions, many of which, like LGMFS, present as non-specific, slow-growing, painless masses. Conditions considered in our patient’s differential diagnosis included Bartholin gland cyst, angiomyofibroblastoma, lipomas, dermatofibrosarcoma protuberans, squamous cell carcinoma, and Bartholin gland adenocarcinoma.

Imaging studies offer limited specificity in diagnosing LGMFS. While Morii et al. (16) and Niu et al. (17) reported the usefulness of 18F-Fluorodeoxyglucose-positron emission tomography (FDG-PET)/computed tomography (CT) in diagnosing LGMFS, the rarity of reported cases limits the generalizability of these findings. An MRI was performed in our case but did not reveal any particularly distinctive features. Establishing uniform diagnostic criteria for LGMFS using only MRI evaluation remains challenging. Nevertheless, including preoperative MRI is crucial in evaluating soft tissue sarcomas’ margins, helping to prevent unnecessary aggressive surgical procedures (5, 18).

The gold standard for diagnosing tumors typically relies on histopathological examination (19). LGMFS must be distinguished from other malignant spindle-cell tumors, such as leiomyosarcoma, malignant fibrous histiocytoma, and spindle-cell metaplastic carcinoma, which are more common in the vulva and share histological similarities with LGMFS. In this case, a preoperative biopsy might have directed initial surgical management toward oncology, potentially preventing the need for a second operation (20, 21). Given the rarity of LGMFS, successful biopsy requires adequate and representative tissue for histological examination, immunohistochemistry, and ancillary molecular studies.

Electron microscopy (EM) can provide detailed structural information at the cellular and subcellular levels. Under electron microscopy, LGMFS displays typical myofibroblastic characteristics, including spindle or stellate tumor cells with abundant microfilaments, microtubules, and collagen fibers, reflecting its fibroblastic properties and active protein synthesis (22). These features help pathologists distinguish LGMFS from other soft tissue tumors, such as fibrosarcoma, leiomyosarcoma, malignant fibrous histiocytoma, synovial sarcoma, and fibromatosis.

Immunohistochemically, LGMFS can be distinguished from other tumors by specific marker expressions. CD34 expression is uncommon in LGMS, whereas angiosarcoma typically demonstrates strong positivity for this endothelial marker (23). LGMFS lacks S100, which helps exclude MPNST and schwannomas (23). α-SMA is focally positive in LGMFS, supporting its myofibroblastic origin, whereas leiomyosarcoma shows widespread SMA positivity. The absence of STAT6 and H3 K27Me3 loss excludes solitary fibrous tumor (SFT), and the lack of p16 and CDK4 rules out dermatofibrosarcoma protuberans (DFSP) (24, 25). Fluorescence *in situ* hybridization (FISH) confirmed no PDGFB (22q13) translocation or COL1A1::PDGFB fusion, which are common in DFSP, and no MDM2 amplification, typically seen in liposarcomas (26). This comprehensive diagnostic approach combining clinical, imaging, immunohistochemical, and genetic findings helped confirm the diagnosis of LGMFS (**Table 1**).

**Table 1:** Test results for the differential diagnosis of LGMFS.

Feature	Description	Comparison with Other Tumors
EM	· Spindle or stellate tumor cells · Abundant microfilaments, microtubules, and collagen fibers · Reflects fibroblastic properties and active protein synthesis	Distinguish LGMFS from fibrosarcoma, leiomyosarcoma, malignant fibrous histiocytoma, synovial sarcoma, and fibromatosis
CD34	· Uncommon in LGMFS	· Strong positivity in angiosarcoma
S100	· Negative in LGMFS	· Positive in MPNST and schwannomas
α-SMA	· Focally positive in LGMFS	· Widespread SMA positivity in leiomyosarcoma
STAT6 and H3 K27Me3	· Both absent in LGMFS	· Present in SFT
p16 and CDK4	· Both absent in LGMFS	· Present in DFSP
FISH	· No PDGFB translocation or COL1A1::PDGFB fusion · No MDM2 amplification	· PDGFB translocation or COL1A1::PDGFB fusion in DFSP · MDM2 amplification in liposarcomas

This table summarizes key features for the differential diagnosis of LGMFS, highlighting electron microscopy findings, immunohistochemical markers, and genetic differences compared to other soft tissue tumors. LGMFS, Low-grade myofibroblastic sarcoma; EM, Electron microscopy; FISH, Fluorescence *in situ* hybridization; SFT, Solitary fibrous tumor; DFSP, Dermatofibrosarcoma protuberans.

Due to the rarity of reported cases, the biological behavior of LGMFS remains poorly understood, and treatment strategies continue to be debated. Reported cases of LGMFS at other anatomical sites typically show slow tumor growth, with recurrent lesions often lacking increased proliferative activity or histological atypia (27). Based on this pattern, the current literature generally recommends wide excision with R0 margins as the preferred treatment (28, 29). A 2 cm margin, as suggested by Kim et al., is often ideal, though this may be adjusted based on tumor location and surrounding structures (7). Preoperative MRI can aid in planning the excision margins, helping to avoid overly aggressive surgery. For vulvar malignancies, partial vulvectomy is appropriate for stage IA tumors, while radical vulvectomy and/or inguinal lymphadenectomy are required for stages IB-III (30). In the two previous cases of vulvar LGMFS, one patient underwent wide local excision with negative margins (13), while the other required a radical local excision (RLE) following an incomplete initial resection due to tissue adhesion. In our case, the initial surgery achieved negative margins; however, after further consultation with the patient, we decided to proceed with an additional RLE to ensure complete excision.

Regarding inguinal lymphadenectomy, it is a standard procedure for vulvar cancers beyond stage IA (30). However, neither of the previous patients required lymphadenectomy, and no recurrence was observed during follow-up. Studies have indicated that vulvar sarcomas primarily metastasize hematogenously (31, 32), with rare lymphatic spread, and the benefits of inguinal lymphadenectomy are minimal (3, 14). Given these findings, we did not perform inguinal lymphadenectomy in our current case.

Radiotherapy and chemotherapy appear to offer no additional therapeutic benefit in the treatment of LGMFS, which are often used as adjunctive treatments for malignant tumors. Large studies, such as the one conducted by Xu et al. (33), have not found substantial evidence supporting the efficacy of those treatments, especially when negative surgical margins are achieved (13, 34, 35). Nonetheless, an individualized approach should be considered, particularly in cases where complete resection is not feasible. In certain instances, patients have shown favorable outcomes with radiotherapy following partial resection, indicating a potential role for adjuvant therapy in specific circumstances. However, the decision to utilize such therapies must be tailored to the individual case, informed by multidisciplinary discussions, and consider critical factors such as tumor size, location, and the patient’s overall health—each of which significantly impacts sarcoma prognosis (36).

In our case, the tumor presented as a localized recurrence without distant metastasis. For this non-pregnant patient in our case, we ultimately opted for a wide local excision with clear margins, deeming it sufficient without the need for additional radiotherapy or chemotherapy. Further comprehensive research is necessary to establish more definitive treatment guidelines despite these considerations.

### 3.3 Pregnancy-specific management: balancing maternal and fetal outcomes

Pregnancy is characterized by increased circulating blood volume, hormonal fluctuations, and an immunosuppressive state. In the perineal region specifically, pregnancy is associated with an increased number of pelvic floor vessels, venous blood obstruction, and lymphatic reflux of the pelvic floor. These physiological changes could potentially promote cancer growth or progression (37, 38). However, in our case of LGMFS, these effects were not prominently observed, as the tumor exhibited only minimal growth, suggesting that its behavior might be more closely related to its intrinsic aggressiveness rather than the physiological changes associated with pregnancy. LGMFS of the vulva during pregnancy presents unique challenges due to the potential impact of mechanical compression of vulvar blood vessels, tumor obstruction of the delivery route, production of cancer-related inflammatory cytokines, or the risk of metastasis to the fetus or placenta (37). A multidisciplinary approach to LGMFS during pregnancy is essential, as the mother, fetus, and malignancy are distinct yet interacting entities. Given the current understanding of LGMFS, delaying treatment until after delivery, as we did in our case, could be reasonable in specific cases, particularly if the tumor exhibits slow growth and lacks aggressive behavior (39). However, this decision must be made cautiously, as the risk of tumor progression during pregnancy cannot be entirely ruled out. A multidisciplinary approach is essential to evaluate the risks and benefits of deferring treatment, with close monitoring throughout pregnancy being critical.

If active treatment is deemed necessary, surgery remains the primary approach. Post-surgical delivery via elective cesarean section may be a prudent choice, as it can help prevent vulvar wound dehiscence and bleeding during and after childbirth (40). However, vaginal delivery can also be considered, particularly if the vulvar wound has healed well after surgery. While the mechanical dilation of the vulva during labor could hypothetically disseminate tumor cells, there is no clear evidence suggesting that vaginal delivery increases the risk of recurrence. Moreover, pregnancy outcomes and fetal mortality/morbidity do not appear to be significantly affected by invasive treatments.

Adjuvant therapies, including radiotherapy and chemotherapy, are typically used with great caution during pregnancy. Most chemotherapeutic agents can be teratogenic, carcinogenic, or mutagenic to the fetus, particularly during the first trimester. As such, chemotherapy during pregnancy is generally considered only after the second trimester (41). Agents such as paclitaxel (Taxol) and platinum-based drugs may have a reduced impact on the fetus during the later stages of pregnancy but should still be used cautiously, taking fetal development into account. Radiotherapy is extremely limited during pregnancy, especially in abdominal regions, as it can cause fetal growth restriction, malformations, miscarriage, or congenital defects (42). In all, radiotherapy and chemotherapy are typically reserved for situations where the tumor is unresectable or rapidly progressing and are considered only when necessary (40, 43). In cases of the patient wishing to continue the pregnancy strongly, clinicians often face the challenging dilemma of balancing maternal and fetal health during treatment decisions (44). As no previous cases of LGMFS in pregnancy have been reported, there is limited research and guidance on managing this rare scenario. However, considering the slow-growing and locally invasive nature of LGMFS, as well as the clinical experience provided in our case, a strategy of observation and monitoring during pregnancy—followed by surgical excision after delivery—appears to be a feasible approach (45). This approach, of course, requires multidisciplinary collaboration and individualized management. Key factors include the patient’s clinical condition, gestational age, fetal health, tumor staging, and the patient’s treatment preferences. Generally, if LGMFS shows no signs of progression, adjuvant therapies should be postponed until after delivery, if necessary, to minimize fetal risks. Larger studies are needed to establish the best treatment strategies for this rare and challenging condition.

### 3.4 Long-term prognosis and follow-up strategy

Given the rarity of LGMFS, especially in the vulva, long-term prognostic data remain limited. However, emerging evidence from population-based studies and case series suggests that LGMFS is a low-grade malignancy characterized by indolent behavior, a relatively favorable prognosis, and a low rate of distant metastasis (<5%) (3). Reported 5-year overall survival rates approach 80%, with disease-specific survival reaching up to 100% in some low-risk cohorts (7, 46, 47). Despite its low metastatic potential, LGMFS poses a significant risk of local recurrence, with reported recurrence rates ranging from 20.8% to 38%, typically occurring within 12–24 months postoperatively (47, 48).

Several factors have been identified as prognostically relevant. Achieving negative surgical margins (R0 resection) is the most important factor for reducing recurrence (7, 47). Tumors ≥5 cm in size, those located in anatomically complex regions (e.g., head and neck, pelvis), or those with high mitotic activity (>6/10 HPF) are more likely to recur. Our case achieved long-term disease-free survival following a radical excision with a 2 cm margin, consistent with recommendations in existing literature.

Standard follow-up protocols for soft tissue sarcomas recommend physical examination and imaging every 6 months for the first 5 years, followed by annual assessments thereafter. In high-risk cases—such as those with positive surgical margins or prior recurrence—closer surveillance (every 3–4 months) is advisable during the first 2–3 years (49, 50). MRI or CT of the primary site is preferred for local monitoring.

Individualized follow-up plans are essential. Tumors located in deep or less accessible areas may warrant extended surveillance (≥10 years) (51). Patient education also plays a crucial role, enabling early recognition of symptoms suggestive of recurrence. Overall, wide excision with adequate margins and tailored long-term monitoring remains the foundation for successful management of LGMFS.

## 4 Conclusion

LGMFS of the vulva, although rare, can present significant diagnostic and management challenges, particularly during pregnancy. This case highlights the successful management of vulvar LGMFS with a multidisciplinary approach, involving conservative monitoring during pregnancy followed by surgical excision after delivery. The tumor showed minimal growth during pregnancy, suggesting that LGMFS may behave indifferently to physiological changes associated with gestation. Radical local excision with clear margins, without the need for inguinal lymphadenectomy or adjuvant therapies, proved effective in achieving long-term disease-free survival, aligning with prior reports of vulvar LGMFS management. Given the low malignant potential and slow growth of LGMFS, a strategy of observation during pregnancy followed by surgical intervention is feasible. Long-term individualized follow-up is also essential to ensure early detection of recurrence and to improve clinical outcomes. Further research is needed to refine treatment protocols for such rare cases.

## Data Availability

Publicly available datasets were analyzed in this study.
